# Facile Synthesis of PdO.TiO_2_ Nanocomposite for Photoelectrochemical Oxygen Evolution Reaction

**DOI:** 10.3390/molecules28020572

**Published:** 2023-01-06

**Authors:** Amna Altaf, Manzar Sohail, Ayman Nafady, Rashid G. Siddique, Syed Shoaib Ahmad Shah, Tayyaba Najam

**Affiliations:** 1 Department of Chemistry, School of Natural Sciences, National University of Sciences and Technology, Islamabad 44000, Pakistan; 2 Chemistry Department, College of Science, King Saud University, Riyadh 11451, Saudi Arabia; 3 School of Physical Sciences, University of Adelaide, Adelaide 5005, Australia; 4 School of Chemistry and Environmental Sciences, Shenzhen University, Shenzhen 518060, China; 5 Institute of Chemistry, The Islamia University of Bahawalpur, Bahawalpur 63100, Pakistan

**Keywords:** photoelectrochemical water splitting, sol–gel synthesis, oxygen evolution reaction, photocatalytic activity

## Abstract

The rapid depletion of fossil fuels and environmental pollution has motivated scientists to cultivate renewable and green energy sources. The hydrogen economy is an emerging replacement for fossil fuels, and photocatalytic water splitting is a suitable strategy to produce clean hydrogen fuel. Herein, the photocatalyst (PdO.TiO_2_) is introduced as an accelerated photoelectrochemical oxygen evolution reaction (OER). The catalyst showed significant improvement in the current density magnitude from 0.89 (dark) to 4.27 mA/cm^2^ (light) during OER at 0.5 V applied potential. The as-synthesized material exhibits a Tafel slope of 170 mVdec^−1^ and efficiency of 0.25% at 0.93 V. The overall outcomes associated with the photocatalytic activity of PdO.TiO_2_ demonstrated that the catalyst is highly efficient, thereby encouraging researchers to explore more related catalysts for promoting facile OER.

## 1. Introduction

Energy shortage, global warming, and fossil fuel depletion are the major problems of the modern world. These issues endanger the survival of humans as well as restrict social development. The need of the hour is to develop some renewable energy sources [1]. Due to its enormous abundance and 71% coverage of the Earth’s surface, water is the ideal substrate for the conversion of solar energy into fuel. In an ideal system, the water-splitting reaction would be catalyzed by a semiconducting photoelectrode, and the resultant H_2_ would be collected and stored to be used as fuel in the future. In water splitting, the following half reactions take place:

4H^+^_(aq)_ + 4e^−^   2H_2(g)_    E^o^ = 0 Vs NHE (1)

2H_2_O_(l)_     O_2(g)_ + 4H^+^ + 4e^−^     E^o^ = 1.23 V (2)

2H_2_O_(l)_     O_2(g)_ + 2H_2(g)_     E^o^ = −1.23 V (3)

The oxygen evolution reaction (OER), as shown in Equation (2), is an anodic reaction and it is a rate-limiting reaction because it utilizes four electrons to form O_2_. So, the overall efficiency of water splitting depends on OER, and a significant effort has been dedicated to synthesizing OER photocatalysts [2]. Similarly, several catalysts have been developed for the electrochemical water splitting [3,4,5]. Both electrochemical and photochemical water splitting have some advantages as well as disadvantages. Combining the advantages of both reactions, photoelectrochemical (PEC) water splitting is one of the best alternatives [3]. This is one of the sustainable and green techniques for oxygen generation in sunlight [4]. For better PEC results, the valence bands and conductive bands of the synthesized material span the redox potential of water [5,6]. 

Different materials have been investigated for PEC water splitting, i.e., metal sulfides [7], oxides [8], and hydroxides [9]. Due to its non-toxicity, higher resistance to corrosion, and chemical stability, TiO_2_ can act as photocatalyst for water splitting. However, TiO_2_ does not show good results due to electron hole recombination and a wide band gap. To overcome this issue, heterojunctions and composite with Pt, Ni, Ag, and Au have been synthesized [10]. Furthermore, noble metal doping in titanium dioxide enhanced the photocatalytic oxygen evolution reaction (OER) [11]. 

Among all these materials, the noble metal palladium (Pd) is considered to be a versatile [12] and efficient catalyst for (OER) in electrochemical water splitting, and its electrocatalytic behavior is continuously being explored [13,14,15]. Moreover, it is more cost-effective and readily available than Pt [15]. Pd is not much explored for the PEC applications. J. Kim et al. loaded the PdO catalyst on the BiVO_4_ for the oxygen evolution reaction. The introduced catalyst improved the stability and current efficiency and reduced surface charge recombination [16]. However, the composite of Pd into TiO_2_ had been rarely explored for the PEC water splitting; more specifically, the OER performance has never been explored. It can be anticipated that a composite of Pd in TiO_2_ may efficiently improve OER performance in PEC water splitting.

Herein, we have, for the first time, introduced the PdO.TiO_2_ catalyst via facile synthesis strategy to accelerate OER during PEC water splitting. The PdO.TiO_2_ is prepared via the simple sol–gel method, which showed a significant improvement in current from 0.89 (dark) to 4.27 mA/cm^2^ (Light) during OER at 0.5 V applied potential (Vs. Ag/AgCl/3M KCl). The photocatalytic activity of PdO.TiO_2_ is attributed to the unique characteristic composite formation between the PdO and TiO_2_. The heterojunction has reduced the band gap to 2.7 eV, enhancing the catalytic activity.

## 2. Results

**Figure 1:**
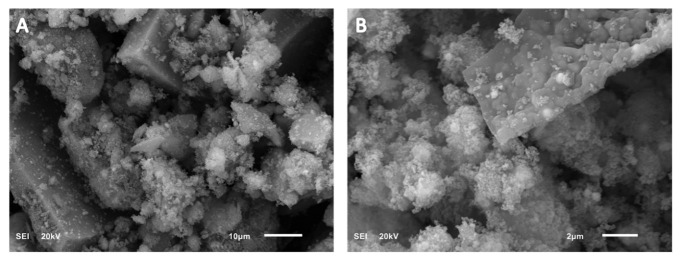
SEM images of PdO.TiO_2_ at two magnifications: (**A**) 10 µm and (**B**) 2 µm.

**Figure 2:**
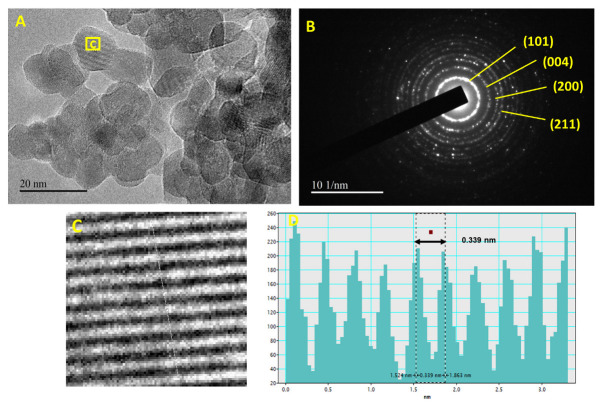
(**A**) TEM images of the pseudo dumbbell shape PdO.TiO_2_ nanoparticles at 20 nm; (**B**) SAED pattern; (**C**) inverse FFT image; (**D**) profile graph of inverse FFT image.

The as-synthesized catalyst was investigated by using the scanning electron microscope (SEM) and the transmission electron microscope (TEM). The SEM images have shown a porous catalyst (Figure 1), and not much useful information regarding morphology was attained with the help of SEM. Due to this reason, the synthesized photocatalyst morphology was further explored using TEM analysis. TEM analysis has shown the very small particle size of the PdO.TiO_2,_ around 20 nm, and all particle sizes are less than 50 nm (Figure 2). TEM images have shown the unique morphology of the photocatalyst. The nanocomposite of the PdO.TiO_2_ appeared as a pseudo-dumbbell shape. This geometrical arrangement can provide more active sites for the PEC reaction and better performance for water splitting.

High-resolution transmission electron microscopy (HR-TEM) images in Figure 2A shows the grain boundary and crystal lattice fringes of the particles. Interplanar spacing of 0.339 nm, calculated by indexing of crystal lattice fringes corresponds to (101) plane of PdO.TiO_2_, matches closely with d spacing 0.34 nm calculated from p-XRD pattern of (101) plane. TEM also confirms the formation of PdO.TiO_2_. Furthermore, the SAED pattern of PdO.TiO_2_ shown in Figure 2B appeared in a concentric circles form representing the (101), (004), (200), and (211) having d-spacing closely matching the d-spacing calculated from p-XRD of PdO.TiO_2_.

**Figure 3:**
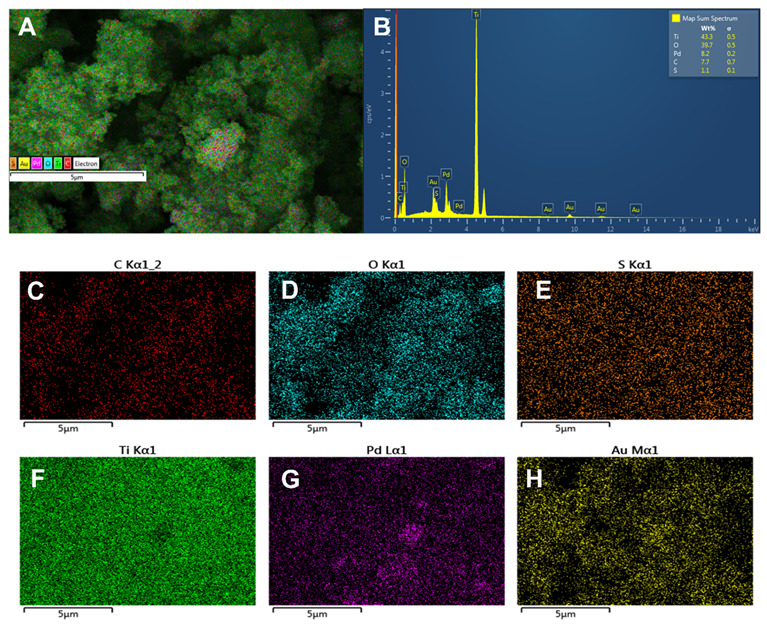
(**A**) EDX analysis of the PdO.TiO_2_; (**B**) elemental and compositional analysis; (**C**–**H**) mapping analysis.

For the development of an efficient photocatalyst for the PEC water splitting, the composite between Pd and TiO_2_ has been synthesized. The composite formation can be confirmed with the help of the EDX analysis. The EDX analysis showed the Pd peak with the shape response of Ti and O, confirming the presence of Pd on the photocatalyst. The Pd composition in the catalyst was around 8.2%, while the Ti and O were 43.3 and 39.7%, respectively Figure 3B. Furthermore, the mapping study revealed that PdO.TiO_2_ had been uniformly formed, see Figure 3C–H. The uniform distribution demonstrated that the synthesizing process efficiently synthesizes the PdO.TiO_2_ catalyst. 

**Figure 4:**
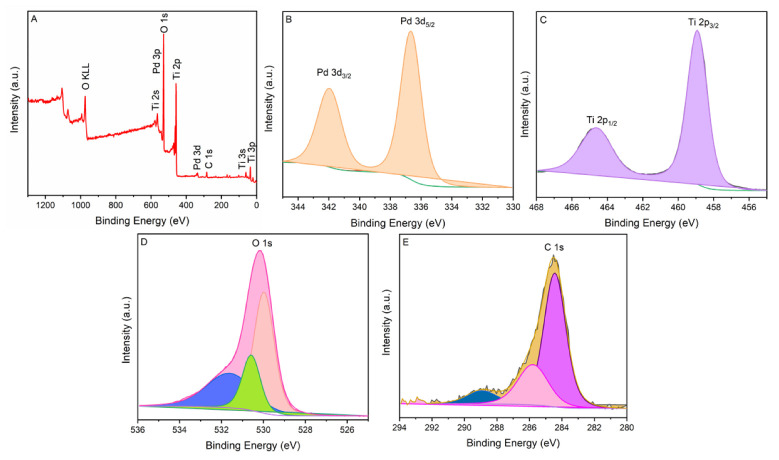
High resolution XPS spectra of the PdO.TiO_2_: (**A**) survey scan; (**B**) Pd 3d_5/2_ and Pd 3d_3/2_ states; (**C**) Ti 2p_1/2_ and Ti 2p_3/2_; (**D**) O 1s; (**E**) C1s reference peak.

The XPS spectroscopy explored the information of the bonding composition and electronic state of PdO.TiO_2_. Figure 4A demonstrates the survey scan, and this scan exhibits peaks of C, O, Ti, and Pd. No extra peaks have been observed depicting the high purity of the as-synthesized sample. The Pd XPS spectrum in Figure 4B displayed the spin orbit doublet of Pd, suggesting that Pd exhibits two valence states Pd^2+^ and Pd^4+^, slightly distorted peaks of 3d_5/2_ and 3d_3/2_, and energy levels at 336.8 and 341.9 eV, respectively. The binding energy difference of the Pd 3d_5/2_ and 3d_3/2_ was found 5.1 eV. The titanium spectra generally displayed the two well-separated peaks at 458.6 eV and the 464.4 eV, corresponding to Ti 2p_3/2_ and Ti 2p_1/2_ [17]. However, the composite of PdO.TiO_2_ causes the blue shift, and the peaks Ti 2p_3/2_ and Ti 2p_1/2_ were shifted to 459.2 and 465.1 eV, respectively, as shown in Figure 4C. The blue shift in the catalyst was found at 0.6 2p_3/2_ and 0.7 eV (2p_1/2_). The binding energy difference of the composite shows Ti peaks at 5.9 eV. The significant change in Ti 3p binding energy indicates that the composite of Pd and TiO_2_ was successfully formed. An O 1s peak of the PdO.TiO_2_ appeared at a binding energy of 530.1 eV. This peak is deconvoluted into three peaks having binding energies of 529.9 eV, 530.5 eV, and 531.6 eV. Figure 4D,E shows that the carbon peak is divided into three peaks located at 284.4 eV, 285.8 eV, and 288.9 eV. The carbon peak appears due to the unintentionally added carbon during sample preparation. However, this appeared peak of carbon is used as a standard peak. 

**Figure 5:**
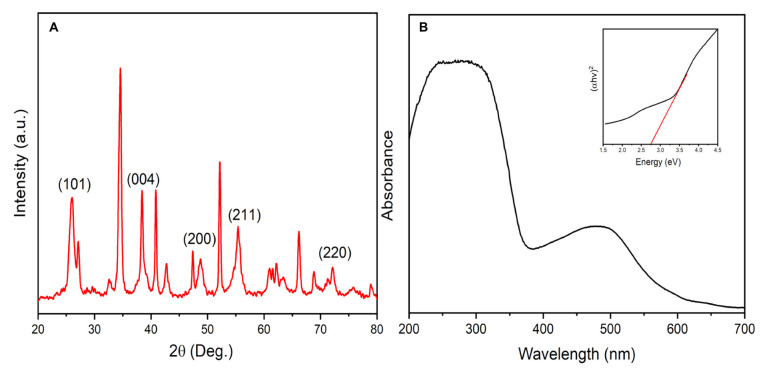
(**A**) p-XRD characterization of the PdO.TiO_2_; (**B**) UV-Vis spectra of PdO.TiO_2_.

Furthermore, the synthesized PdO.TiO_2_ was evaluated by powder X-ray Diffraction (p-XRD) to study the crystallinity and composition of the nanocatalyst. The p-XRD study revealed that after heating at 500 °C the observed catalyst phase was PdO.TiO_2_, seen in Figure 5A. In the p-XRD of the PdO.TiO_2_ nanocomposite the diffraction peaks were observed at 25.9˚, 38.4°, 48.7°, 56.24°, corresponding to the anatase phase of the TiO_2_, and these diffraction peaks can be assigned to crystallographic planes (101), (004), (200), and (211) [18]. The peaks were slightly shifted which might be due to the composite formation (DB card No. 01-075-1537). The p-XRD peaks appeared at 40.7°, 47.5°, 68.7°, and 82.6° which possibly demonstrated the face-centered cubic faces of Pd (111), Pd (200), Pd (220), and Pd (311) planes, respectively [15,19]. The shifting of p-XRD peaks of TiO_2_ [18] and the appearance of the PdO peaks demonstrate that a PdO and TiO_2_ nanocomposite has been formed. 

To investigate the optical properties of the as-synthesized material, UV-Vis absorption spectra has been recorded. As shown in Figure 5B, PdO.TiO_2_ exhibited two absorption bands at 273 nm and 485 nm, which corresponds to a bandgap of 2.7 eV.

**Figure 6:**
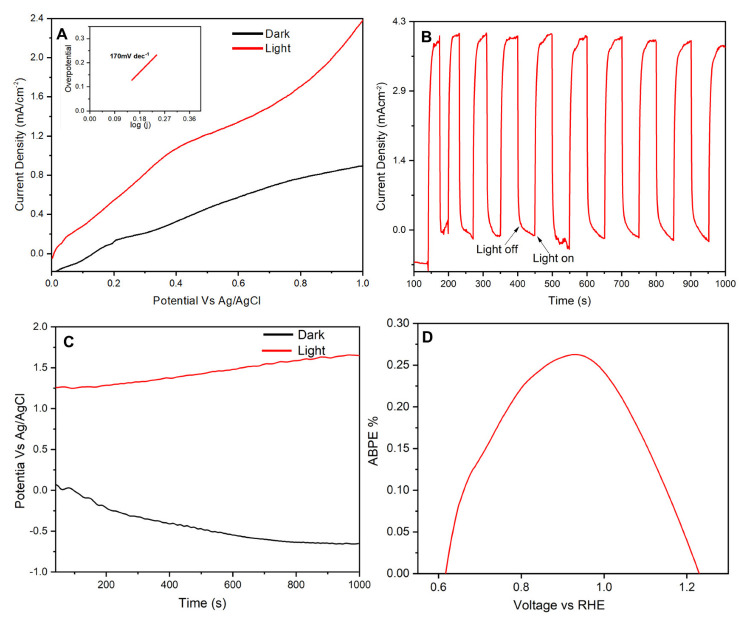
(**A**) LSV scan in the presence and absence of light (inset is the Tafel slope); (**B**) CA chopping at 0.5 V; (**C**) stability; (**D**) photocatalyst efficiency.

Synthesized PdO.TiO_2_ was explored for the photoelectrochemical splitting of water. The as-synthesized composition was found to be highly photochemically active. For photocatalytic activity investigation, the FTO-coated working electrode was exposed to one Sunlight solar simulator, and it has shown the capability to split water in 0.5 M Na_2_SO_4_ at an applied potential. The photocatalyst acts as a photoanode and displays the OER. To investigate the catalyst’s PEC activity, LSV was scanned from 0.0 to +1.0 V under dark and light, respectively. Upon illumination, the current sharply increased from 0.8 mA/cm^−2^ to 2.4 mA/cm^−2^ as shown in Figure 6A. The huge increment in the current on exposure to light is evident that it can prove an excellent catalyst for OER. For evaluation of photocatalyst efficiency, a Tafel plot has been drawn from LSV curves. The Tafel plot as shown in an inset of Figure 6A illustrates that the material has a Tafel slope value of 170 mVdec^−1^.The comparatively lower slope value is attributed to higher PEC activity. Chronoamperometry (CA) was performed under chopped solar light illumination. Upon illumination, the current density increased to 4.27 mA/cm^−2^ as shown in Figure 6B. The stability of the photoelectrocatalyst was also investigated under dark and light at 0.5 V as shown in Figure 6C. The catalyst demonstrated a stable behavior both in the dark and in the presence of light and an almost constant current behavior was observed. This behavior established that PdO.TiO_2_ catalyst remained stable upon exposure to sunlight. 

As a matter of fact, the contact between Pd and TiO_2_, due to the formation of heterojunction and moderate bandgap of composite, leads to better charge separation and lower electron–hole recombination, thereby resulting in excellent PEC performance of the as-prepared PdO.TiO_2_ photoanode. This significantly increased current density can be attributed to better charge separation. Thus, this phase of palladium exhibits remarkable characteristics for PEC water splitting.

Furthermore, the efficiency of the photocatalyst has been calculated by photo to current efficiency (ABPE). ABPE is calculated by using the following formula [20].

ABPE (%) = (J (1.23 − V _bias_) V)/P_in_ ∗ 100 (4)

The ABPE estimated for PdO.TiO_2_ is 0.25% at 0.93 V as shown in Figure 6D. 

## 3. Materials and Methods

### 3.1. Chemicals

Titanium isopropoxide (TTIP), palladium nitrate dihydrate, tetrahydrofuran (THF), and triflic acid (TFC) were obtained from Sigma-Aldrich (Shanghai, China) and used without any further purification.

### 3.2. Synthesis of Palladium Titanate

**Figure 7:**
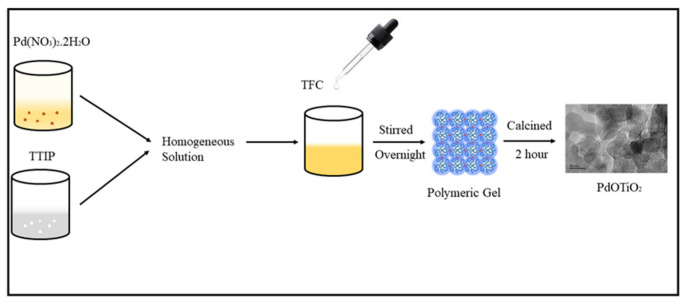
Schematic diagram for synthesis of catalyst.

Pd–Ti-based nanocomposites have been synthesized by mixing palladium nitrate dihydrate and titanium isopropoxide in THF, then THF polymerization was initiated by adding 4–5 drops of TFC, the same scheme was followed as reported by our research group [19]. An amount of 0.0075 M titanium isopropoxide and 0.0075 M palladium nitrate dihydrate was added in 25 mL THF at room temperature. Then, 4–5 drops of TFC were added for THF polymerization. Solution was stirred overnight so that THF polymerized slowly. When polymeric gel formed, the products were calcined at 500 °C for 2 h. After pyrolysis, the as-synthesized material was cooled and stored at room temperature. Synthesis scheme is shown in Figure 7. 

### 3.3. Fabrication of Electrode

Electrode was fabricated by drop casting on fluorine tin oxide (FTO)-coated glass. FTO was cleaned by sonicating in acetone, ethanol, and finally in deionized water, and dried at 70 °C for 30 min. Then, 1 mg of catalyst and a few drops of nafion were dispersed in 2 mL of ethanol. The solution was sonicated for 20 min, and the ink was coated on FTO glass preheated at 50 °C by using a micropipette. Finally, the coated electrodes were dried in vacuum at 50 °C.

### 3.4. PEC Measurement

PEC water splitting was undertaken on Reference 3000, Gamry electrochemical Workstation (Warminster, PA, USA) by using a conventional three electrode system. The FTO-coated glass was used as working electrode, Ag/AgCl/3M KCl as reference electrode, and platinum wire (Pt) as counter electrode in a quartz cell containing 0.5 M Na_2_SO_4_ as electrolyte. A 300 W Xenon lamp was used as the source for visible light illumination.

### 3.5. Characterizations

Elemental and morphological analysis was carried out by scanning electron microscope (SEM) (SEM), with an energy dispersive X-ray (EDX) detector (HITACHI S-4800, Bancroft St. Toledo, OH 43606, USA). Powdered X-ray diffraction (p-XRD) was carried out to study the crystallinity of the material by DRON-8 X-ray diffractometer (Bourevestnik, St. Petersburg, Russia). Cu Kα radiations of 1.54 Å wavelength was used. X-ray photoelectron spectroscopy XPS (Thermo-VG Scientific, ESCALAB250, Qxi microprobe spectrometer, Waltham, MA, USA) was used, with a monochromatic Al Kα X-ray source. Ultraviolet–visible (UV–vis) absorption spectroscopy was performed at room temperature using a PerkinElmer Lambda 35 UV–vis spectrophotometer (Akron, OH, USA).

## 4. Conclusions

In summary, we reported a novel nanocomposite of PdO.TiO_2_, synthesized by a facile method. The as-synthesized PdO.TiO_2_ catalyst gave rise to a current density of 4.27 mA/cm^−2^. This study demonstrates that a porous dumbbell-shaped catalyst can be produced by heterojunction of TiO_2_ with Pd. The channels in the dumbbell-shaped catalyst aid in the electron transfer process and increase reaction kinetics. In addition, while anatase TiO_2_ has a band gap of 3.2 eV, Pd doping has reduced the band gap to 2.7 eV, thereby enhancing the catalyst’s activity for OER. This study proves that Pd can be used as an efficient material for PEC water splitting.

## Data Availability

Data can be provided on request.
